# Chronic tubal ectopic pregnancy with undetectable serum β-hCG mimicking a large ovarian endometrioma: A case report

**DOI:** 10.1016/j.crwh.2026.e00814

**Published:** 2026-04-25

**Authors:** Laiqah Al-Dhaheri, Ahlam Al-Kholani

**Affiliations:** a Department of Obstetrics and Gynecology, 21 September University for Medical and Applied Sciences, Sana'a, Yemen; b Department of Obstetrics and Gynecology, Faculty of Medicine and Health Sciences, Amran University, Amran, Yemen

**Keywords:** Ectopic pregnancy, Chronic ectopic pregnancy, Negative β-hCG, Adnexal mass, Diagnostic challenge, Salpingo-oophorectomy

## Abstract

Ectopic pregnancy is usually associated with detectable serum β-human chorionic gonadotropin levels. A rare case of chronic tubal ectopic pregnancy with undetectable beta human chorionic gonadotrophin (β-HCG) that mimicked a large ovarian endometrioma is reported. A 34-year-old multiparous woman presented with prolonged abnormal uterine bleeding and chronic pelvic pain following a 45-day delay in menstruation. Imaging revealed a large complex left adnexal mass measuring 12 × 10 × 7 cm, highly suggestive of ovarian endometrioma. Surgical exploration demonstrated a densely adherent adnexal mass requiring left salpingo-oophorectomy. Histopathological examination confirmed chronic tubal ectopic pregnancy. This case highlights that ectopic pregnancy should remain in the differential diagnosis of adnexal masses even when serum β-hCG is undetectable.

## Highlights

• Chronic ectopic pregnancy may present with undetectable serum β-hCG levels.
• Large adnexal hematomas can mimic ovarian endometrioma on imaging.
• A negative β-hCG test result does not completely exclude ectopic pregnancy.
• Histopathology remains the definitive diagnostic method.

## 1 Introduction

Chronic ectopic pregnancy is a rare form of ectopic gestation characterized by low trophoblastic activity and atypical clinical presentation [1]. It may present as a pelvic mass and mimic other gynecological conditions such as ovarian endometrioma. Serum β-hCG levels may be low or even undetectable, making the diagnosis challenging. A case of chronic tubal ectopic pregnancy presenting as a large adnexal mass with undetectable serum β-hCG is reported.

## 2 Case Presentation

A 34-year-old multiparous woman presented with chronic left-sided pelvic pain and prolonged abnormal uterine bleeding. She reported a 45-day delay in menstruation followed by continuous vaginal bleeding lasting approximately two months, associated with progressively worsening pelvic pain.

Her obstetric history included five previous term vaginal deliveries. She reported regular menstrual cycles prior to the current episode. There was no history of intrauterine device use, hormonal contraception, previous ectopic pregnancy, pelvic inflammatory disease, or prior pelvic surgery.

A single quantitative serum β-human chorionic gonadotropin (β-hCG) measurement performed one day prior to surgery was negative (<5 IU/L).

**Fig. 1:**
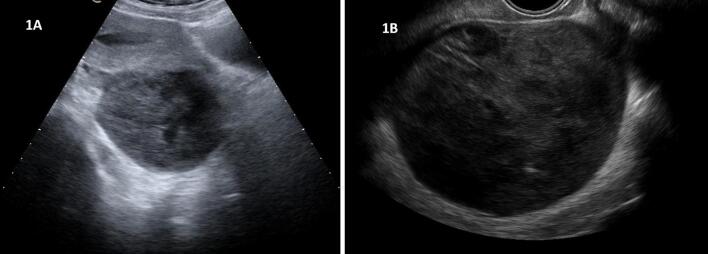
Radiological findings. (A) Transabdominal ultrasound: a large, complex left adnexal mass with heterogeneous echoes, mimicking an endometrioma. (B) Transvaginal ultrasound: Detailed view of the 12 × 10 × 7 cm mass oriented toward the left mesovarian region.

Transvaginal and transabdominal ultrasound demonstrated a large complex left adnexal cystic mass with heterogeneous internal echoes. No intrauterine gestational sac was visualized. The uterus appeared normal in size and morphology. Findings were initially suggestive of ovarian endometrioma (Fig. 1A-B).

Pelvic magnetic resonance imaging (MRI) further characterized the lesion as a large complex cystic mass located posterior to the uterus and oriented toward the left adnexal region, measuring approximately 12 × 10 × 7 cm, demonstrating heterogeneous signal intensity with internal hemorrhagic components.

Imaging interpretation favored a complex ovarian cyst, most consistent with endometrioma.

Given persistent pain, prolonged bleeding, and the significant size of the lesion, surgical management was undertaken.

At laparotomy, a large left adnexal mass was identified, densely adherent to the posterior uterine wall and adjacent pelvic structures, with extensive fibrotic adhesions involving the sigmoid colon. An intraoperative consultation with the general surgery team was obtained to ensure safe adhesiolysis and bowel integrity. The tubo-ovarian anatomy was markedly distorted, and the ovarian tissue was inseparable from the mass. Consequently, a left salpingo-oophorectomy was performed to achieve complete excision and hemostasis. The other pelvic organs appeared grossly normal.

**Fig. 2:**
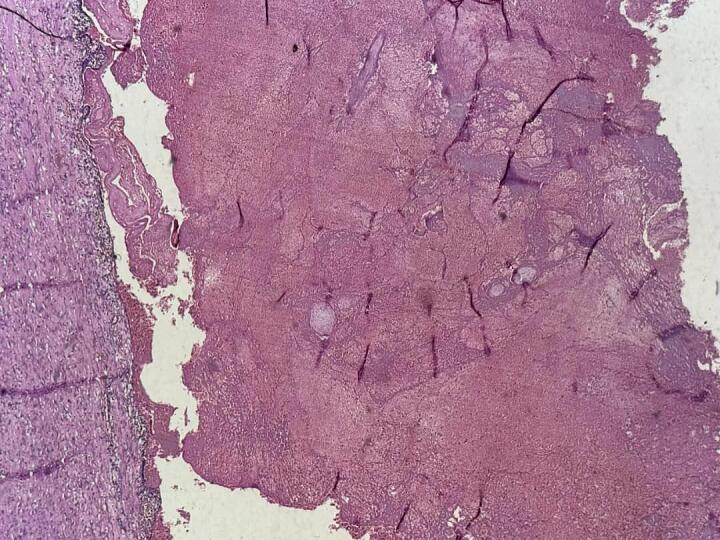
Histopathology - low power. Photomicrograph showing the fallopian tube wall and a lumen filled with organized hemorrhage and fibrinoid material (H&E, ×40).

**Fig. 3:**
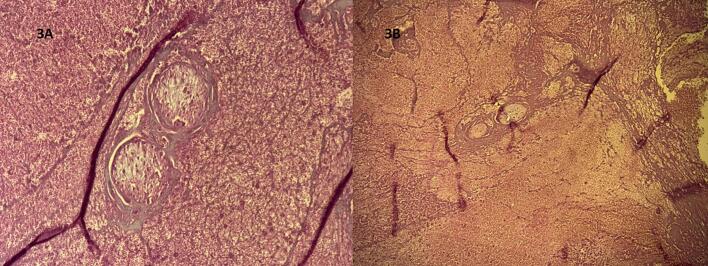
A–B: Histopathology - high power. High magnification reveals necrotic “ghost” chorionic villi, confirming a chronic ectopic pregnancy despite the negative serum β-hCG (H&E, ×400).

Histopathological examination revealed a fallopian tube distended with organized blood clots and multiple chorionic villi within the lumen, consistent with chronic tubal ectopic pregnancy. No evidence of granulomatous inflammation, molar changes, or malignancy was identified (Fig. 2, Fig. 3).

The postoperative course was uneventful, and the patient recovered well. The patient was counseled regarding future fertility and the small risk of recurrent ectopic pregnancy. She was advised to seek early medical evaluation in any future pregnancy for confirmation of intrauterine gestation. Contraceptive counseling was also provided, and the patient opted for barrier contraception.

## 3 Discussion

Ectopic pregnancy (EP) is typically diagnosed through a combination of clinical presentation, serial measurements of serum β-human chorionic gonadotropin (β-hCG), and transvaginal ultrasonography [1], [2]. In contemporary clinical practice, a negative quantitative β-hCG level is generally considered sufficient to exclude an ongoing ectopic pregnancy. However, rare atypical presentations challenge this assumption.

The vast majority of ectopic pregnancies occur within the fallopian tube [3]. Chronic ectopic pregnancy represents a distinct clinical and pathological entity characterized by repeated minor bleeding from the implantation site, progressive trophoblastic degeneration, and organization of intratubal hematoma [4]. As trophoblastic viability declines, hormonal production diminishes, potentially resulting in very low or even undetectable serum β-hCG levels. This mechanism likely explains the negative quantitative β-hCG observed in the present case despite definitive histopathological identification of chorionic villi within the fallopian tube.

Clinically, chronic ectopic pregnancy often presents with prolonged abnormal uterine bleeding and pelvic pain rather than acute abdominal symptoms. In this case, the patient experienced 45 days of amenorrhea followed by two months of irregular uterine bleeding accompanied by persistent pelvic pain, a pattern consistent with chronic ectopic implantation rather than acute rupture.

Radiologically, chronic ectopic pregnancy may closely mimic other adnexal pathologies. Organized hematoma, hemolyzed blood products, and inflammatory adhesions can produce imaging features resembling ovarian endometrioma, hemorrhagic cyst, tubo-ovarian abscess, or even neoplasm [4], [5]. In this patient, both ultrasound and MRI findings favored a diagnosis of endometrioma due to the presence of a large complex cystic lesion with heterogeneous internal echoes and dense adhesions. The absence of detectable β-hCG further reduced clinical suspicion for ectopic pregnancy.

Although cases of low or declining β-hCG in ectopic pregnancy have been described, completely negative quantitative β-hCG in histologically confirmed ectopic pregnancy remains rare, although several case reports have described ruptured or chronic ectopic pregnancies occurring despite undetectable serum β-hCG levels [4], [5], [6], [7]. This case emphasizes that β-hCG testing, while highly sensitive, is not infallible. Clinicians should maintain a high index of suspicion in reproductive-aged women presenting with adnexal masses and abnormal bleeding, particularly when the clinical history includes recent amenorrhea.

This case underscores the importance of maintaining clinical suspicion for ectopic pregnancy in reproductive-aged women presenting with adnexal masses and abnormal uterine bleeding, even when serum β-hCG levels are undetectable.

Definitive diagnosis in such atypical presentations relies on histopathological examination. The identification of chorionic villi within the fallopian tube remains the gold standard for confirming ectopic pregnancy [1]. Awareness of unusual presentations is essential to avoid misdiagnosis and delays in appropriate surgical management.

## 4 Conclusion

Chronic ectopic pregnancy may present with atypical clinical and biochemical findings, including undetectable serum β-hCG levels. It can mimic ovarian endometrioma on imaging studies. Clinicians should maintain a high index of suspicion when evaluating adnexal masses in reproductive-aged women.

## Contributors

Laiqah Al-Dhaheri contributed to patient care, conception of the case report, acquiring and interpreting the data, undertaking the literature review and drafting the manuscript.

Ahlam Al-Kholani contributed to patient care, acquiring and interpreting the data and revising the article critically for important intellectual content.

Both authors approved the final submitted manuscript.

## Patient consent

Written informed consent was obtained from the patient for publication of this case report and accompanying images.

## Provenance and peer review

This article was not commissioned and was peer reviewed.

## Declaration of generative AI and AI-assisted technologies in the writing process

During the preparation of this work the authors used generative AI tools only for language editing and grammar improvement. After using these tools, the authors reviewed and edited the content as needed and take full responsibility for the content of the publication.

## Funding

No funding from an external source supported the publication of this case report.

## Declaration of competing interest

The authors declare that they have no competing interest regarding the publication of this case report.
